# CD146 positive human dental pulp stem cells promote regeneration of dentin/pulp-like structures

**DOI:** 10.1007/s13577-017-0198-2

**Published:** 2018-01-08

**Authors:** Mikiko Matsui, Tomoko Kobayashi, Takeo W. Tsutsui

**Affiliations:** 1 Department of Pharmacology, The Nippon Dental University School of Life Dentistry at Tokyo, 1-9-20 Fujimi, Chiyoda-ku, Tokyo, 102-8159 Japan; 2 Department of Periodontology, Graduate School of Medical and Dental Sciences, Tokyo Medical and Dental University (TMDU), 1-5-45 Yushima, Bunkyo-ku, Tokyo, 113-8510 Japan

**Keywords:** CD146, Human dental pulp stem cells, Mineralization, Transplantation, Regenerative therapy

## Abstract

CD146 and STRO-1 are endothelial biomarkers that are co-expressed on the cellular membranes of blood vessels within human dental pulp tissue. This study characterized the percentage of dentin-like structures produced by CD146-positive (CD146^+^) human dental pulp stem cells (DPSCs), compared with their CD146-negative (CD146^−^) counterparts. DPSC populations were enriched using magnetic-activated cell sorting (MACS), yielding CD146^+^ and CD146^−^ cells, as well as mixtures composed of 25% CD146^+^ cells and 75% CD146^−^ cells (CD146^+/−^). Cell growth assays indicated that CD146^+^ cells exhibit an approximate 3–4 h difference in doubling time, compared with CD146^−^ cells. Cell cycle distributions were determined by flow cytometry analysis. The low percentage of CD146^+^ cells’ DNA content in G_0_/G_1_ phase were compared with CD146^−^ and non-separated cells. In contrast to CD146^−^ and non-separated cells, prompt mineralization was observed in CD146^+^ cells. Subsequently, qRT-PCR revealed high mRNA expression of *CD146* and *Alkaline phosphatase* in mineralization-induced CD146^+^ cells. CD146^+^ cells were also observed high adipogenic ability by Oil red O staining. Histological examinations revealed an increased area of dentin/pulp-like structures in transplanted CD146^+^ cells, compared with CD146^−^ and CD146^+/−^ cells. Immunohistochemical studies detected dentin matrix protein-1 (DMP1) and dentin sialophosphoprotein (DSPP), as well as human mitochondria, in transplanted DPSCs. Co-expression of CD146 and GFP indicated that CD146 was expressed in transplanted CD146^+^ cells. CD146^+^ cells may promote mineralization and generate dentin/pulp-like structures, suggesting a role in self-renewal of stem cells and dental pulp regenerative therapy.

**Electronic supplementary material:**

The online version of this article (10.1007/s13577-017-0198-2) contains supplementary material, which is available to authorized users.

## Introduction

Mesenchymal stem cells (MSCs) have been isolated from several human tissues; their ability for self-renewal and multilineage differentiations suggests potential for regenerative cell therapy. MSCs are capable of generating tissues needed for wound repair, and can be differentiated into osteocytes, chondrocytes, adipocytes, and odontoblasts [1, 2]. These cells have been characterized in vitro and tested in transplantation applications, where they have demonstrated therapeutic potential for the repair and regeneration of damaged tissues.

Multiple studies have indicated that bone-marrow (BM) skeletal stem cells, also termed stromal or mesenchymal stem cells (BM-MSCs), have distinctive surface markers, such as CD146, CD105, CD106, and STRO-1 [3–6]. BM-MSC markers have been used as stem-cell markers for periodontal ligament and dental pulp tissue (e.g., STRO-1, CD105, and CD146) [5–7]. Notably, CD146 is a stem cell and endothelial marker that is co-expressed with STRO-1 on cells that comprise the outer layer of blood vessel walls [6].

CD146 is a cell adhesion molecule and integral membrane glycoprotein at the intercellular junction, which was originally identified as a tumor marker for melanoma [4, 8]. Baksh et al. reported high expression of CD146 in human umbilical cord perivascular cells [9]; CD146-positive perivascular cells demonstrate functional and gene-expression profiles that are similar to MSCs [10]. Furthermore, CD146 is up-regulated on highly proliferative cells and enables trilineage differentiation in BM-MSCs [11]. Therefore, CD146 has been suggested as a marker for MSCs from adult and fetal organs.

CD146-positive BM-MSCs have been shown to possess high migration ability and stromal cells supporting hematopoiesis [3]. In addition, CD146-positive cells that expressed Angiopoietin-1 have been shown to be relevant to hematopoietic microenvironment [12]. CD146 is expressed in DPSCs with alpha-smooth muscle actin and/or the pericyte-associated antigen 3G5—this indicates that DPSCs reside in the perivascular niche [6].

DPSCs were first isolated from normal human extracted third molars and showed a similar immunophenotype as BM-MSCs in vitro [2]. In the study, both DPSCs and BM-MSCs that were transplanted into immunocompromised mice successfully generated either dentin/pulp-like structures or bone tissue-like structures. The another report of cDNA microarray analysis has also revealed similar gene expression in DPSCs and BM-MSCs [13]. DPSCs can differentiate into multiple cell lineages (osteogenesis, adipogenesis, and chondrogenesis) [14, 15]; notably, this ability extends to neural differentiation as well [14, 16]. These reports demonstrate that DPSCs have a self-renewal and differentiation capacity that is similar to BM-MSCs.

Previous cellular isolation methods that use CD146 as a marker were mainly performed on BM-MSCs [11, 17] and human umbilical cord MSCs [9, 18]. Tavangar et al. performed an in vitro analysis of DPSCs that were isolated with the CD146 marker [19]. However, there is not yet a clear understanding of CD146-positive cellular migration within dentin/pulp-like structures in vivo. Furthermore, the CD146-positive cell population has not been demonstrated for its ability to perform mineralization in vitro, or its ability to form dentin-like structures in vivo.

In this study, we analyzed whether CD146-positive human DPSCs (CD146^+^ cells) promote regeneration of dentin/pulp-like structures. We separated DPSCs into CD146^+^ cells and CD146-negative cells (CD146^−^ cells), then compared both populations with non-separated cells via cellular morphology, cellular growth, and the cell cycle assay. We also prepared a mixture of 25% CD146^+^ cells and 75% CD146^−^ cells (referred to as CD146^+/−^ cells). We analyzed high mineralization ability of CD146^+^ cells by alizarin red S staining and qRT-PCR, compared with non-separated cells, CD146^−^ cells, and CD146^+/−^ cells. Oil red O staining indicated high adipogenic ability of CD146^+^ cells. Transplanted CD146^+^ cells into immunocompromised mice also demonstrated their abilities to generate dentin/pulp-like structures, compared with CD146^−^ cells and CD146^+/−^ cells. Immunohistochemistry studies were used to confirm the successful transplantation of human-derived cells and to assay the formation of dentin-like structures.

## Materials and methods

### Cell culture

Human DPSCs were obtained from the third molar of an 11-year-old patient. The informed consent was taken from parent/LAR of the patient under approved by the Committee of Ethics, the Nippon Dental University School of Life Dentistry at Tokyo. The pulp tissue was digested in Ca^2+^- and Mg^2+^-free phosphate-buffered saline (PBS; pH 7.4) containing 3 mg/ml collagenase type I (Sigma-Aldrich, Tokyo, Japan) and 4 mg/ml dispase (Invitrogen, Grand Island, NY, USA) for 1.5 h at 37 °C. Single-cell suspensions were cultured according to published methods [20], in minimum essential medium alpha (α-MEM; Gibco/Life Technologies, Tokyo, Japan) supplemented with 20% fetal bovine serum (FBS; Nichirei Biosciences, Tokyo, Japan), 100 µM l-ascorbic acid phosphate magnesium salt *n*-hydrate (Wako Pure Chemical Industries, Osaka, Japan), 2 mM l-glutamine (Gibco/Life Technologies), 0.22% NaHCO_3_ (Wako), 100 units/ml penicillin, and 100 µg/ml streptomycin (Gibco/Life Technologies) at 37 °C with 5% CO_2_.

### Isolation of CD146^+^ cells and CD146^−^ cells

DPSCs were separated into CD146^+^ and CD146^−^ cell groups using magnetic-activated cell sorting (MACS; Miltenyi Biotec Inc., Bergisch Gladbach, Germany). Cells were reacted with FcR Blocking Reagent and CD146 MicroBeads (CD146 MicroBead Kit; Miltenyi Biotec Inc.) according to the manufacturer’s instructions, then loaded onto a MACS^®^ Column. LS Columns were used for CD146^+^ cell selection, while LD Columns were used for CD146^−^ cell selection.

### CD146^+^ cells’ rate assay by flow cytometry

Cells in each group of CD146^+^ cells, CD146^−^ cells, and non-separated cells were incubated with PE-conjugated human CD146 antibodies (130-097-939, Miltenyi Biotec Inc.) or PE-conjugated mouse IgG1 isotype control antibodies (130-098-845, Miltenyi Biotec Inc.) for 10 min at 4 °C. After washing with autoMACS Running Buffer, 5 × 10^5^ cells were analyzed using Guava^®^ easyCyte flow cytometers (Merck Millipore, Darmstadt, Germany).

### Cell growth assay

Cell proliferation and doubling times of individual CD146^+^ cells, CD146^−^ cells, and non-separated cells were determined by a logarithmic growth curve with five timepoints (0, 1, 2, 3, and 4 days). Cells were plated at approximately 1.19 × 10^3^ cells/cm^2^. Cell counts are presented as the mean ± SD from three dishes (*n* = 3) per time point.

### Cell cycle assay

Cells in each group of CD146^+^ cells, CD146^−^ cells, and non-separated cells were seeded at the same concentration as the cell growth assay, then harvested after 2 or 3 days. Cells were fixed by cold 70% ethanol (− 20 °C) for ≥ 12 h. After washing with PBS, 2 × 10^5^ cells were stained with 200 µl propidium iodide (PI; Guava^®^ Cell Cycle Reagent, Merck Millipore) for 30 min at room temperature in the dark. Samples were analyzed on Guava^®^ easyCyte flow cytometers.

### Mineralization assay

Non-separated cells, CD146^+^ cells, CD146^−^ cells, and CD146^+/−^ cells were plated at 1 × 10^4^ cells/well in 24-well plates, and cultured until 80–100% confluent. Then mineralization was induced in α-MEM supplemented with 10% FBS, 100 µM l-ascorbic acid phosphate magnesium salt *n*-hydrate, 2 mM l-glutamine, 100 units/ml penicillin and 100 µg/ml streptomycin, 10 mM sodium β-glycerophosphate *n*-hydrate (Wako), and 10 nM dexamethasone (Wako) for up to 21 days. Cells were harvested at 3, 7, 10, 14, and 21 days after induction; all cells were fixed with 4% paraformaldehyde (Wako) and stained with 2% alizarin red S (Sigma-Aldrich).

### Quantitative reverse transcription polymerase chain reaction (qRT-PCR)

Total RNA was extracted from individual cell groups (non-separated cells, CD146^+^ cells, CD146^−^ cells, and CD146^+/−^ cells) using the RNeasy Mini Kit (Qiagen, Hilden, Germany). cDNA was synthesized with the High Capacity RNA-to-cDNA Kit (Thermo Fisher Scientific, Waltham, MA, USA) according to the manufacturer’s instructions. qRT-PCR was performed using TaqMan^®^ Gene Expression Master Mix (Applied Biosystems, Foster City, CA, USA) on a StepOnePlus™ System (Thermo Fisher Scientific). The primers used for qRT-PCR were specific for VIC-conjugated *β*-*actin* (4326315E; internal control), FAM-conjugated *CD146* (Hs00174838_m1), *Alkaline phosphatase* (Hs01029144_m1), and *Osteocalcin* (Hs01587814_g1). Data were analyzed on triplicate samples by the StepOne™ Software v2.2.2 (Thermo Fisher Scientific), and presented as relative expression of each gene, compared with non-separated cells at 80–100% confluence.

### Adipogenic differentiation assay

Non-separated cells, CD146^+^ cells, CD146^−^ cells, and CD146^+/−^ cells were plated at 5.1 × 10^4^ cells/well in six-well plates. Cells were cultured in adipogenic induction medium; α-MEM supplemented with 20% FBS, 0.5 mM 3-isobutyl 1-methylxanthine (Sigma-Aldrich), 0.5 μM hydrocortisone (Sigma-Aldrich), 60 μM indomethacin (Sigma-Aldrich), 100 μM ascorbic acid, and 2 mM l-glutamine for up to 14 days. Cells were harvested at 7 and 14 days after induction and stained with Oil red O (Sigma-Aldrich).

### Transplantation and immunohistochemical analysis

CD146^+^ cells were transfected with green fluorescent protein (GFP) by electroporation (Super Electroporator NEPA21; Nepa Gene Co. Ltd., Chiba, Japan). pCMV-EGFP (amplified in the DH5α strain of *Escherichia coli*; 6086-1, BD Biosciences Clontech, Palo Alto, CA) was kindly provided by Kensuke Miki (Yokohama City University). Approximately 2 × 10^6^ cells of either CD146^+^, CD146^−,^ or CD146^+/−^ cells mixed with Ceratite^®^ (NGK Spark Plug Co, Aichi, Japan) were transplanted into immunocompromised beige mice (Crl: NIH-Lyst^bg^Foxn1^nu^Btk^xid^) as previously described [20]. These experiments were performed under approved by the Animal Experiments Committee of The Nippon Dental University School of Life Dentistry at Tokyo and The Nippon Dental University School of Life Dentistry at Tokyo Safety Management Regulations on Genetic Recombination Experiments. The transplants were fixed in 4% paraformaldehyde, decalcified with 10% ethylenediaminetetraacetic acid, and stained with hematoxylin–eosin (H–E). Immunohistochemistry was performed using the primary antibodies against either CD146 (ab75769, 1:250; Abcam, Cambridge, UK), human mitochondria (ab92824, 1:200; Abcam), GFP (2555, 1:500; Cell Signaling Technology, Danvers, MA, USA), dentin matrix protein-1 (HPA037465, 1:50; Sigma-Aldrich), or dentin sialophosphoprotein (HPA036230, 1:50; Sigma-Aldrich). Histostain^®^-SP kits (Invitrogen) were used for biotinylated second antibodies and for HRP enzyme-avidin conjugate incubation, according to the manufacturer’s instructions. Samples were observed with the BIOREVO BZ-9000 microscope (Keyence, Osaka, Japan).

Dentin-like structures on H–E images were surrounded by dotted lines, and the percentage of dentin-like structures area (DSA) was calculated per whole image area using Adobe Photoshop for image analysis (Adobe Photoshop CC; Adobe Systems Inc., San Jose, CA) [21, 22].

## Results

### Isolation of CD146^+^ and CD146^−^ cell groups from DPSCs

**Fig. 1:**
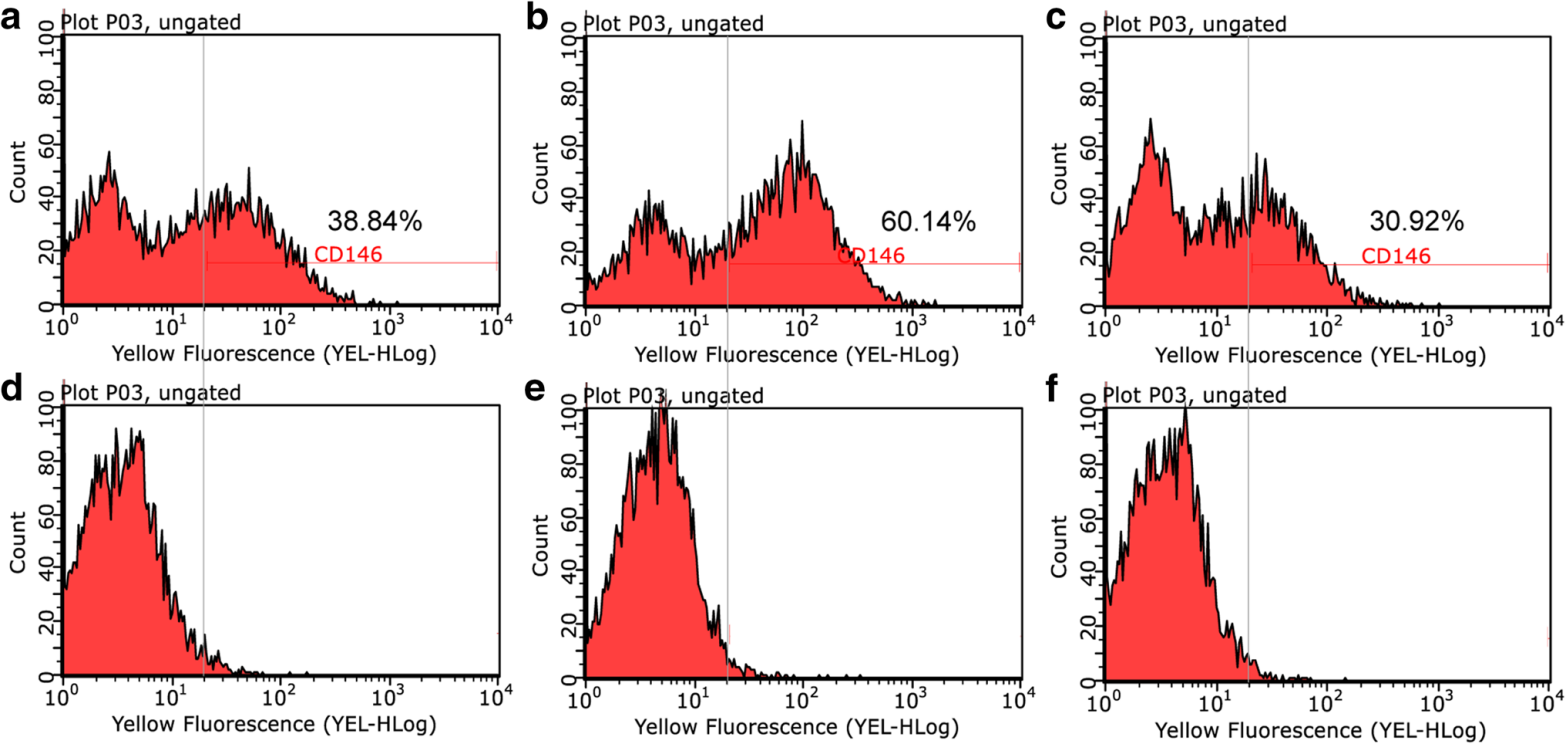
Flow cytometry analysis of the proportion of CD146 in each cell group (**a**–**c**), compared with isotype control (**d**–**f**). CD146 of non-separated (**a**), CD146^+^ (**b**), and CD146^−^ cells (**c**). Isotype control of non-separated (**d**), CD146^+^ (**e**), and CD146^−^ cells (**f**)

The proportion of CD146^+^ cells in each cell subpopulation was assessed by flow cytometry (Fig. 1). The separated subpopulation of CD146^+^ cells included greater proportion of CD146^+^ cells (60.14%) (Fig. 1b), compared with CD146^−^ subpopulation (30.92% CD146^+^ cells) (Fig. 1c) and non-separated cell group (38.84% CD146^+^ cells) (Fig. 1a).

### Cell morphology, cell proliferation, and cell cycle analysis of non-separated, CD146^+^ and CD146^−^ cells

**Fig. 2:**
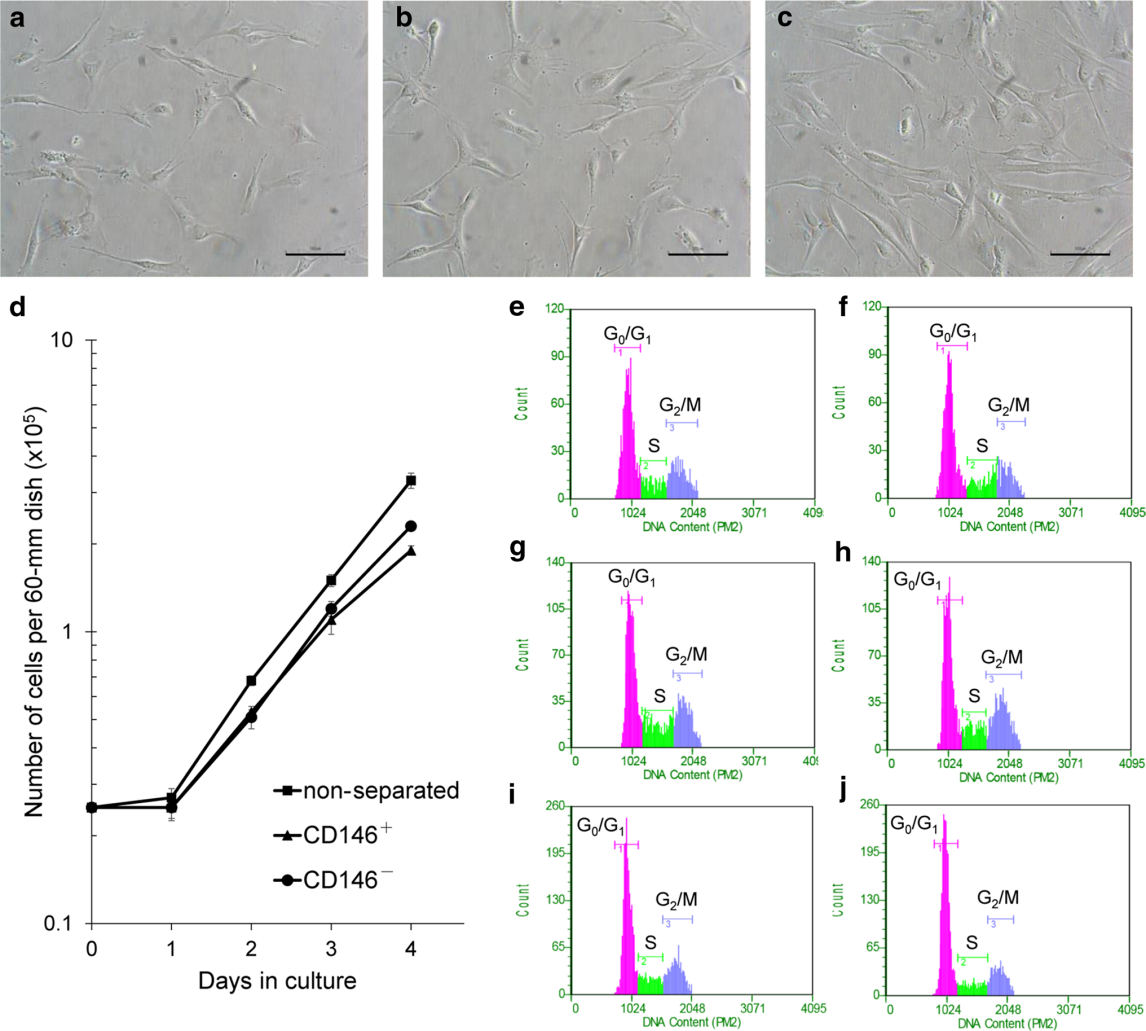
Comparisons of cell morphology, cell growth, and cell cycle among cell groups. Morphology of non-separated (**a**), CD146^+^ (**b**), and CD146^−^ cells (**c**). **d** Cell growth curve of each cell group. Cell numbers are presented as the mean ± SD from three independent experiments. Cell cycle analyses of non-separated (**e**, **f**), CD146^+^ (**g**, **h**), and CD146^−^ cells (**i**, **j**) at 2 day (**e**, **g**, **i**) and 3 day (**f**, **h**, **j**) post-seeding. Scale bar = 100 µm (**a**, **b**, **c**)

Separated groups of CD146^+^ and CD146^−^ cells revealed a similar fibroblast-like morphology as seen in the non-separated cells (Fig. 2a–c). CD146^−^ cells (Fig. 2c) showed a most elongated spindle-like morphology in all cell groups.

Cell proliferation was evaluated by cell growth curve (Fig. 2d) and doubling time (DT). DT calculation and the stage of cell cycle analysis used cells harvested at 2 and 3 day post-seeding. DTs of non-separated, CD146^+^ and CD146^−^ cells were 21.1, 22.9, and 19.5 h, respectively (Table 1). The cell cycle analysis at 2 days indicated a low percentage of CD146^+^ cells in G_0_/G_1_ phase (50.3%), compared with non-separated (57.8%) and CD146^−^ cells (58.9%) (Fig. 2e–j, Table 1), as measured by total DNA content.

**Table 1:** Comparisons of doubling time (hours) and cell cycle distributions (%) among non-separated cells, CD146^+^ cells, and CD146^−^ cells

Sample	Doubling time^a,b^	2 days			3 days		
		G_0_/G_1_^c^	S^c^	G_2_/M^c^	G_0_/G_1_	S	G_2_/M
Non-separated	21.1	57.8	15.1	27.1	60.4	18.4	21.2
CD146^+^	22.9	50.3	22.2	27.5	51.6	15.0	33.4
CD146^−^	19.5	58.9	15.2	25.9	64.3	13.8	21.9

^a^Doubling times are presented as hours

^b^Doubling time = 24 × log2/[log (cell number of 3 days) − log (cell number of 2 days)]

^c^Cell cycle distributions are presented as %

### Comparison of mineralization potential among non-separated, CD146^+^, CD146^−^, and CD146^+/−^ cells

**Fig. 3:**
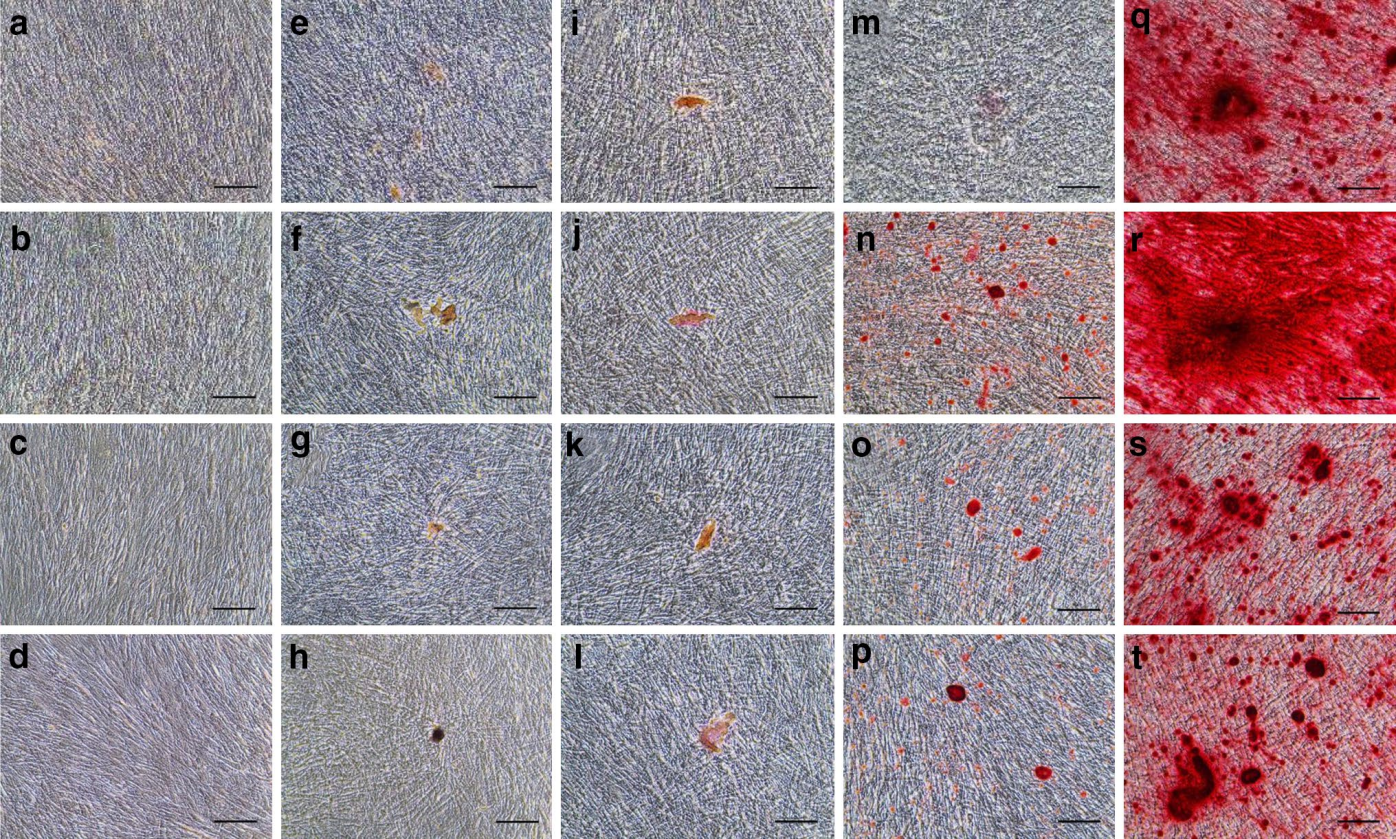
Alizarin red S staining of mineralization potential (**a**–**t**) following induction with differentiation medium for 3 days (**a**–**d**), 7 (**e**–**h**), 10 days (**i**–**l**), 14 days (**m**–**p**), and 21 days (**q**–**t**). Non-separated (**a**, **e**, **i**, **m**, **q**), CD146^+^ (**b**, **f**, **j**, **n**, **r**), CD146^−^ (**c**, **g**, **k**, **o**, **s**), and CD146^+/−^ cells (**d**, **h**, **l**, **p**, **t**). Scale bars = 100 µm (**a**–**t**)

We observed mineralization potential at 3 days (Fig. 3a–d), 7 days (Fig. 3e–h), 10 days (Fig. 3i–l), 14 days (Fig. 3m–p), and 21 days (Fig. 3q–t) after differentiation induction. We compared non-separated (Fig. 3a, e, i, m, q), CD146^+^ (Fig. 3b, f, j, n, r), CD146^−^ (Fig. 3c, g, k, o, s), and CD146^+/−^ cells (Fig. 3d, h, l, p, t). Alizarin red negative staining was observed through 3 day post-induction (Fig. 3a–d). Positive staining in CD146^+^ cells (Fig. 3f) and pseudo-positive staining in non-separated, CD146^−^ and CD146^+/−^ cells (Fig. 3e, g, h), first appeared at 7 day post-induction. By 14 day post-induction, mineralized matrix deposition had significantly increased in CD146^+^ cells (Fig. 3n), compared with non-separated, CD146^−^, or CD146^+/−^ cells (Fig. 3m, o, p). By 21 day post-induction, all cell groups showed a great expansion of mineralization (Fig. 3q–t); notably, CD146^+^ cells were extensively filled with a densely stained matrix and mineralized nodules (Fig. 3r).

### Gene expression of *CD146*, *Alkaline phosphatase* and *Osteocalcin*

**Fig. 4:**
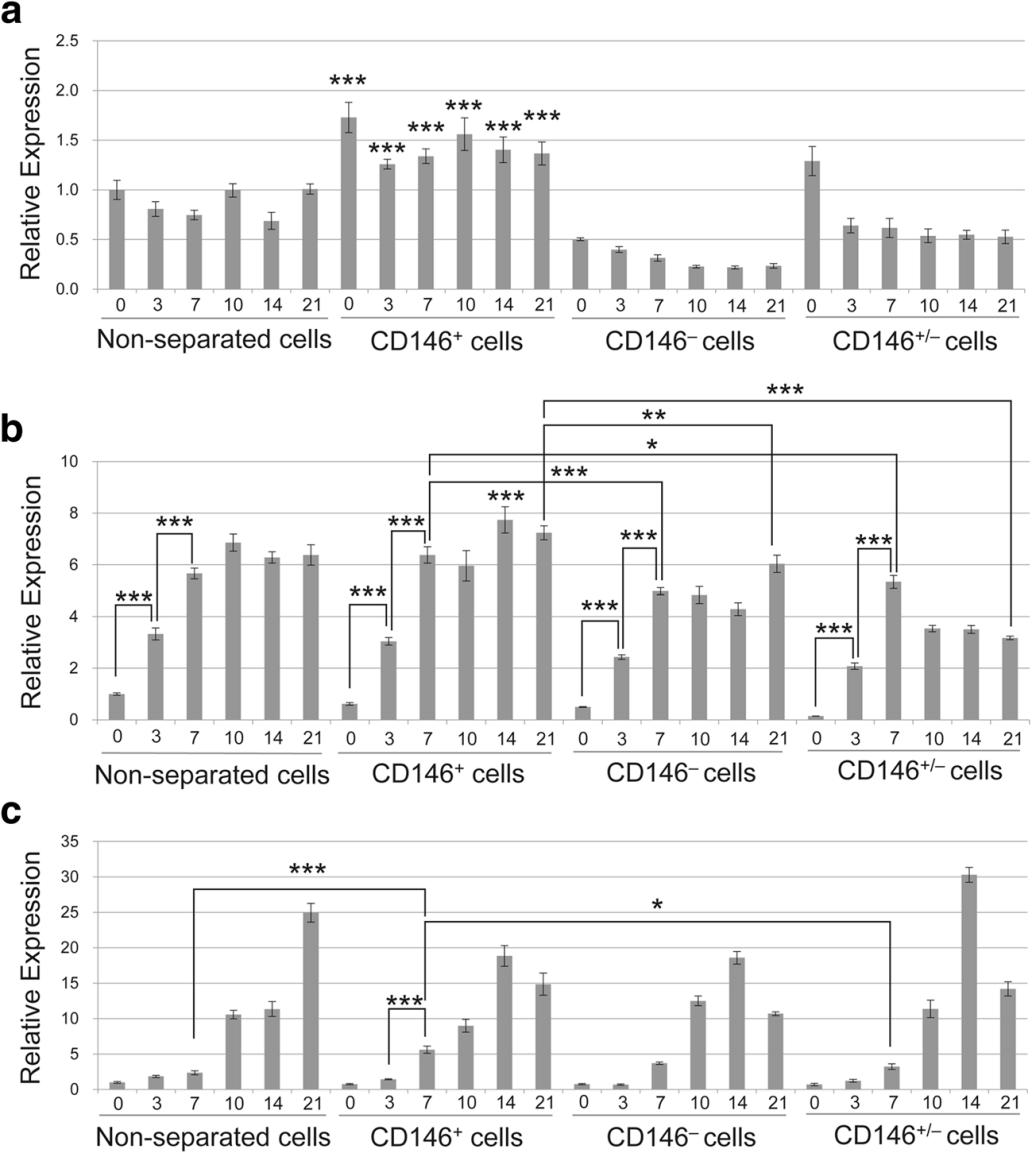
qRT-PCR analysis of *CD146* mRNA (**a**), *Alkaline phosphatase* (*ALP*) mRNA (**b**), and *Osteocalcin* mRNA (**c**) expression in differentiation medium. Each group was analyzed after induction with differentiation medium for 0, 3, 7, 10, 14, and 21 days. All data were compared with non-separated cells at 0 days that were 80–100% confluent. Three statistical analyses were performed using a one-way ANOVA with Tukey’s post-test. The data are expressed as mean ± SD of three tests. *0.01 ≤ *p* < 0.05, **0.001 ≤ *p* < 0.01, ***p < 0.001

We analyzed gene expression at 3, 7, 10, 14, and 21 days (Fig. 4), correlated with observation points of alizarin red staining (Fig. 3). All data are presented as relative expression compared with non-separated cells at 0 days. *CD146* expression was significantly higher in CD146^+^ cells, compared with either non-separated, CD146^−^, or CD146^+/−^ cells from the same time points (Fig. 4a). CD146^−^ cells exhibited significantly lower *CD146* expression through 21 day post-induction, compared with non-separated cells. *Alkaline phosphatase* (*ALP*) in all cell groups exhibited statistically significant increases between 0 and 3 day and between 3 and 7 day post-induction (Fig. 4b). Remarkably, CD146^+^ cells exhibited higher *ALP* expression from 7 through 21 day post-induction, compared with CD146^−^ and CD146^+/−^ cells. *Osteocalcin* expression was not significantly different among non-separated, CD146^−^ and CD146^+/−^ cells through 7 day post-induction (Fig. 4c). However, CD146^+^ cells exhibited statistically significant upregulation of *Osteocalcin* between 3 and 7 day post-induction.

### Comparison of adipogenic potential among non-separated, CD146^+^, CD146^−^ , and CD146^+/−^ cells

**Fig. 5:**
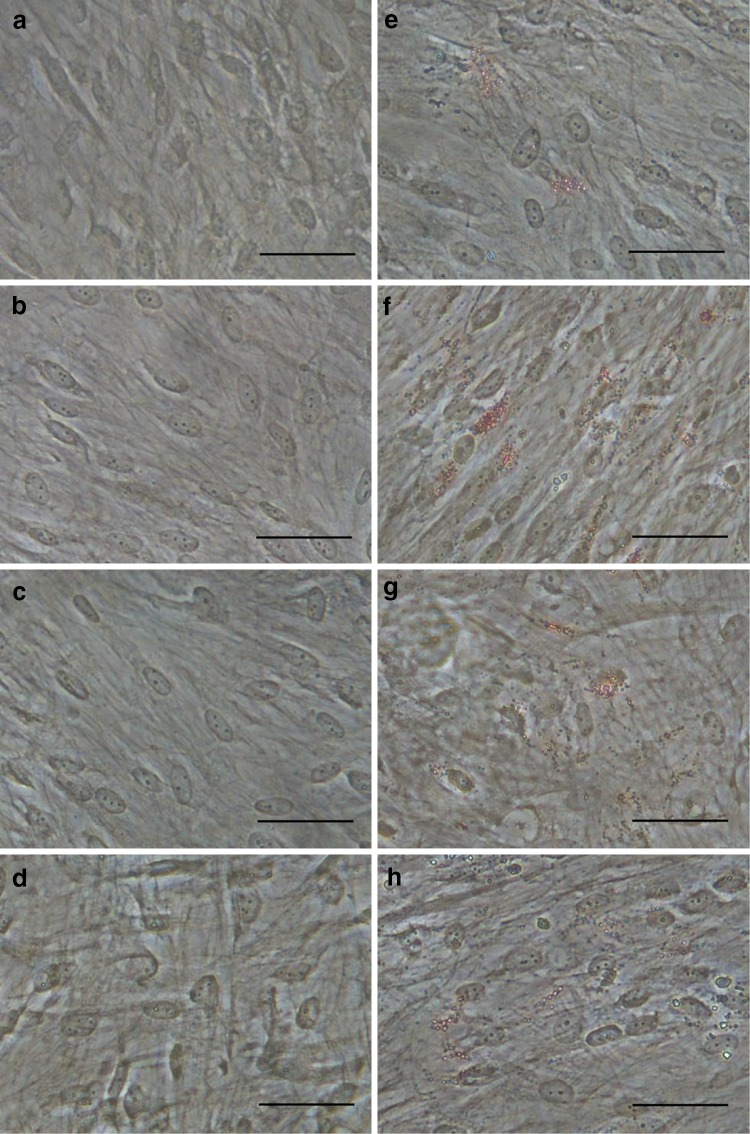
Oil red O staining of adipogenic potential (**a**–**h**) following induction with differentiation medium for 7 days (**a**–**d**) and 14 days (**e**–**h**). Non-separated (**a**, **e**), CD146^+^ (**b**, **f**), CD146^−^ (**c**, **g**), and CD146^+/−^ cells (**d**, **h**). Scale bars = 50 µm (**a**–**h**)

We observed adipogenic potential at 7 days (Fig. 5a–d) and 14 days (Fig. 5e–h) after differentiation induction. Non-separated (Fig. 5a), CD146^+^ (Fig. 5b), CD146^−^ (Fig. 5c), and CD146^+/−^ cells (Fig. 5d) showed negative staining through 7 days. At 14 days, lipid droplet formation was observed in all cell groups (Fig. 5e–h); particularly, CD146^+^ cells showed significantly increased lipid droplets (Fig. 5f).

### Generation of dentin/pulp-like structures in vivo

**Fig. 6:**
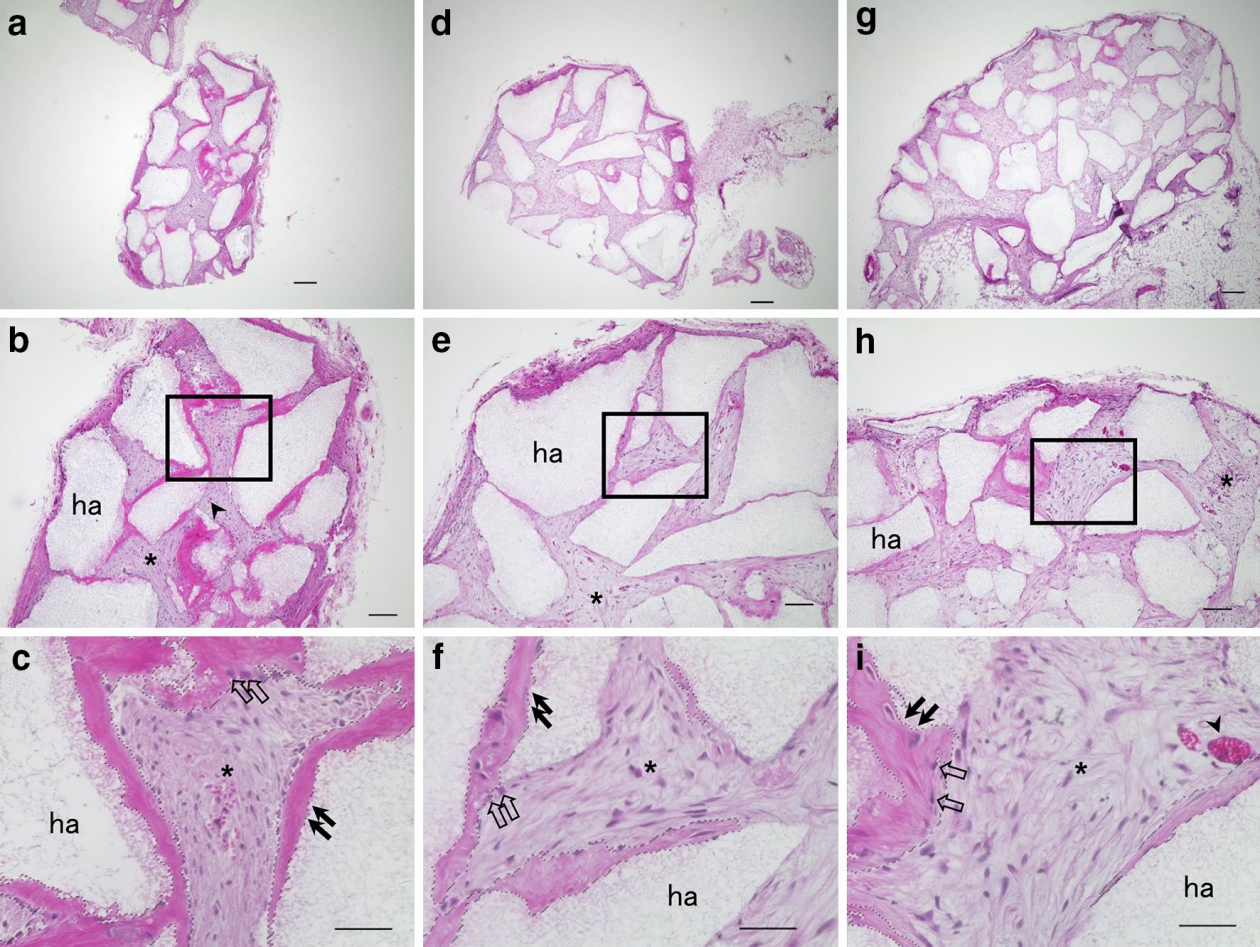
Cross sections are representative of transplanted CD146^+^ (**a**, **b**, **c**), CD146^−^ (**d**, **e**, **f**), and CD146^+/−^ cells (**g**, **h**, **i**). Low magnification of transplants (**a**, **d**, **g**). High magnification of the box region in (**b**, **e**, **h**) shows dentin/pulp-like structures (**c**, **f**, **i**). ha, HA/TCP carriers; asterisk, connective tissue; arrowhead, blood vessels; black arrow, dentin-like structure; open arrow, odontoblast-like cells. Dotted lines (**c**, **f**, **i**) denote area selected for analysis of DSA. Scale bars = 200 (**a**, **d**, **g**), 100 (**b**, **e**, **h**), and 50 µm (**c**, **f**, **i**)

The ability to generate dentin/pulp-like structures was compared among CD146^+^, CD146^−^, and CD146^+/−^ cells (Fig. 6). CD146^+^ cells had formed thick dentin-like and dentin/pulp-like structures (Fig. 6a–c). In contrast, CD146^−^ (Fig. 6d–f) and CD146^+/−^ cells (Fig. 6g–i) had formed fewer dentin-like matrix and pulp-like connective tissue. The DSA in Fig. 6c, f, and i were calculated as 25.9, 12.3, and 13.5%, respectively (Supplemental Table S1). Furthermore, DSA was analyzed in three separate sections from each transplant group (Supplemental Fig. S1, Supplemental Table S1) and all CD146^+^ cell transplants exhibited a high DSA, compared with cell transplants from other groups (Supplemental Fig. S1a–c).

**Fig. 7:**
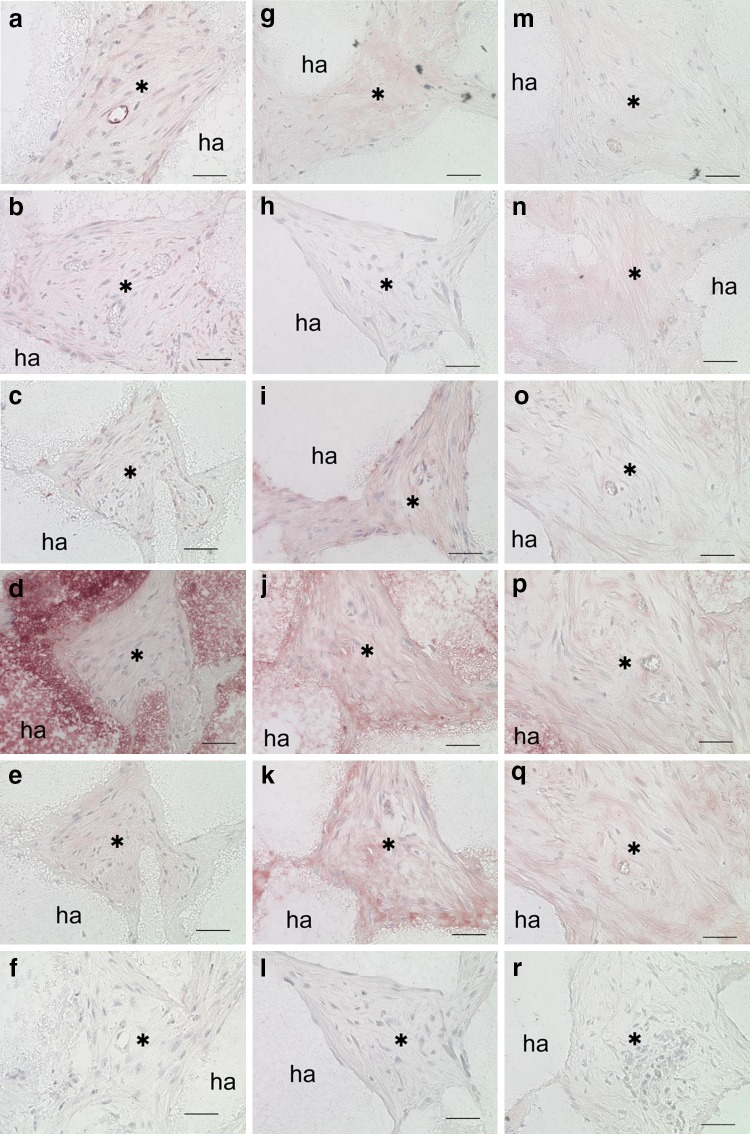
Histological and immunohistochemical analysis of transplantation (**a**–**r**). CD146^+^ (**a**–**f**), CD146^−^ (**g**–**l**), and CD146^+/−^ cells (**m**–**r**). Immunohistochemical staining with antibodies against CD146 (**a**, **g**, **m**), GFP (**b**, **h**, **n**), human mitochondria (**c**, **i**, **o**), DMP1 (**d**, **j**, **p**), DSPP (**e**, **k**, **q**), and no antibody (negative control; **f**, **l**, **r**). ha, HA/TCP carriers; asterisk, connective tissue. Scale bars = 50 µm (**a**–**r**)

Strong expression of both CD146 and GFP was detected in connective tissues harvested from CD146^+^ cell transplants (Fig. 7a, b). CD146^−^ (Fig. 7g) and CD146^+/−^ (Fig. 7m) cell transplants exhibited weak CD146 expression in connective tissues. CD146^+/−^ cell transplants exhibited weak GFP expression in connective tissue (Fig. 7n); on the other hand, CD146^−^ cell transplants were not exhibited GFP (Fig. 7h).

The presence of human mitochondria, DMP1, and DSPP was observed among all three transplants (Fig. 7c–e, i–k, o–q). CD146^+^ cell transplants exhibited uniform expression of these markers within connective tissues (Fig. 7c–e), whereas both CD146^−^ and CD146^+/−^ cell transplants exhibited heterogeneous expression of these markers in connective tissues (Fig. 7i–k, o–q).

## Discussion

The populations of DPSCs that play a critical role in tissue repair have not been fully identified. CD146 is a stem-cell marker that is associated with angiogenic, neurogenic, and mineralization abilities [8, 23]. In our study, MACS-separated CD146^+^ cells exhibited a greater rate (60.14%) of CD146 expression, compared with CD146^−^ (30.92%) and non-separated cells (38.84%). Olbrich et al. reported the use of MACS to isolate osteoprogenitor cells, and detected an approximately 45% expression of mesenchymal stem cell antigen-1, in MACS-separated osteoprogenitor cells [24]. Another study involving MACS separation detected a 71.41% rate of CD146 expression in CD146^+^ BM-MSCs [17]. Isolation efficiency of CD146^+^ cells is highly variable across samples, as indicated by three separate STRO-1^+^/CD146^+^ periodontal ligament cell harvests: these harvests exhibited 50.00, 58.77, and 93.38% rates of CD146 expression [25]. Consequently, we regarded the purity of CD146^+^ cells in this study as an effective positive rate for analyzing their stem-cell properties.

Stem cells are quiescent during homeostasis, and are regulated by a balance between proliferation and differentiation during the repair of injured tissues [26, 27]. In our study, the doubling time of CD146^+^ cells was 22.9 h; a low percentage of CD146^+^ cells were in G_0_/G_1_ phase, compared with non-separated and CD146^−^ cells. Absent in melanoma-2 (AIM2), a protein involved in CD146 expression, induced G_2_/M cell phase arrest [28]. Thus, we suspect that the low percentage of G_0_/G_1_ phase cells in our samples of CD146^+^ cells may indicate G_2_/M cell phase arrest for regulation of a balance between proliferation and differentiation.

CD146^+^ MSCs, sometimes referred to as pericytes, have exhibited a high osteoblastic potential [23, 29, 30]; thus, CD146^+^ cells may promote differentiation and/or mineralization. We analyzed the mineralization ability of four groups of cells (non-separated, CD146^+^, CD146^−^, and CD146^+/−^) in vitro. The proportions within the CD146^+/−^ cell group (25% CD146^+^ cells and 75% CD146^−^ cells) were designed to independently validate data from the non-separated cell group; thus, we utilized flow cytometry data from CD146^+^ cells (60.14% CD146-positive) and CD146^−^ cells (30.92% CD146-positive) [CD146^+^ cells (60.14%) × 25% + CD146^−^ cells (30.92%) × 75% = 38.23%]; this percentage is nearly identical to the proportion of CD146-positive cells (38.84%) that we measured in the non-separated cell group. Mineralization by CD146^+^ cells promoted both alizarin red-positive staining and early upregulation of *Osteocalcin* mRNA expression. qRT-PCR of CD146^+^ cells also revealed high expression of *CD146* and *ALP* compared with other cell groups. Therefore, CD146^+^ cells showed high mineralization ability in agreement with the previous studies [23, 29, 30]. Furthermore, Oil red O staining indicated high adipogenic ability of CD146^+^ cells, compared with non-separated, CD146^−^, and CD146^+/−^ cells. This result also supports the evidence of high differentiation capacity of CD146^+^ cells.

CD146^+^ cells may play an important role in generating DPSC niche via regulation of angiogenesis. We evaluated the abilities of CD146^+^, CD146^−^, and CD146^+/−^ cells to generate dentin/pulp-like structures. Transplanted CD146^+^ cells generated clear dentin/pulp-like structures. In contrast, CD146^−^ and CD146^+/−^ cells generated fewer dentin-like structures and pulp-like connective tissues. In addition, GFP was transfected into CD146^+^ cells, and these transfected cells co-expressed GFP and CD146. Strong CD146- and GFP-positive staining was found in connective tissues harvested from CD146^+^ cell transplants. Staining for human mitochondria, DMP1, and DSPP was observed in transplants of each cell group. DMP1 and DSPP are odontoblast-specific markers, and their presence confirms the secretion of dentin components [31]. Human mitochondria, DMP1, and DSPP were detected uniformly in connective tissues of CD146^+^ cell transplants. This indicated that transplanted CD146^+^ cells played an active role in the formation of dentin/pulp-like structures. In contrast, CD146^−^ and CD146^+/−^ cell transplants exhibited heterogeneous expression of these markers (human mitochondria, DMP1, and DSPP), implying a low differentiation ability for both cell groups.

In our study, the CD146^−^ cell group was determined to be a heterogeneous cell population, including 30.92% CD146-positive cells that were insufficiently separated by MACS. The low percentage of CD146-positive cells in the CD146^−^ cell group might induce a fewer dentin/pulp-like structures. CD146^+/−^ cells showed different ability of mineralization and regeneration from non-separated cells, despite the nearly identical constitution to CD146-positive cell percentage in non-separated cell group. This suggests that the difference of some factors implicated in microenvironment of CD146^+/−^ cells compared with non-separated cells. The advantage of the in vivo assay used in this study was the ability to determine the role of CD146^+^ and CD146^−^ cells in the generation of dentin-like structures and in the formation of the dentin/pulp-like complex in the target cells.

Our results demonstrate that isolated CD146^+^ cells are a putative stem-cell population with a high differentiation ability. CD146^+^ cells showed an increase in differentiation ability compared with CD146^−^ and CD146^+/−^ cells, both in vitro and in vivo. Moreover, CD146^+^ cells may play important roles in the formation of dentin/pulp-like structures and their essential microenvironment. Although the regenerative and differential property of DPSCs using this separation cells for in vitro and in vivo assay of various other marker of stem cells/progenitor cells, our findings suggests that this assay system may be useful for assessment of regeneration potential in other stem/progenitor cells. In addition, this assay system may be employed to detect new markers of stem/progenitor cells. Finally, our study suggests the use of CD146^+^ DPSCs in dental pulp regenerative therapy. However, further mechanistic analyses of the ability for CD146^+^ cells to self-renewal are required for effective utilization of DPSCs in regenerative therapy.

## Electronic supplementary material

Below is the link to the electronic supplementary material.

**Supplemental Figure S1** Dentin-like structures analysis of CD146^+^ (**a**, **b**, **c**), CD146^−^ (**d**, **e**, **f**), and CD146^+/−^ cells (**g**, **h**, **i**). Dotted line surrounds generated dentin-like structures area (DSA). Scale bars = 50 µm (**a**–**i**)

Supplementary material 2 (DOCX 17 kb)
