# Supplementary figures and images for: CD146 positive human dental pulp stem cells promote regeneration of dentin/pulp-like structures
Source: Hum Cell. 2018 Jan 8;31(2):127–38. doi: 10.1007/s13577-017-0198-2 (PMC5852189; doi:10.1007/s13577-017-0198-2)

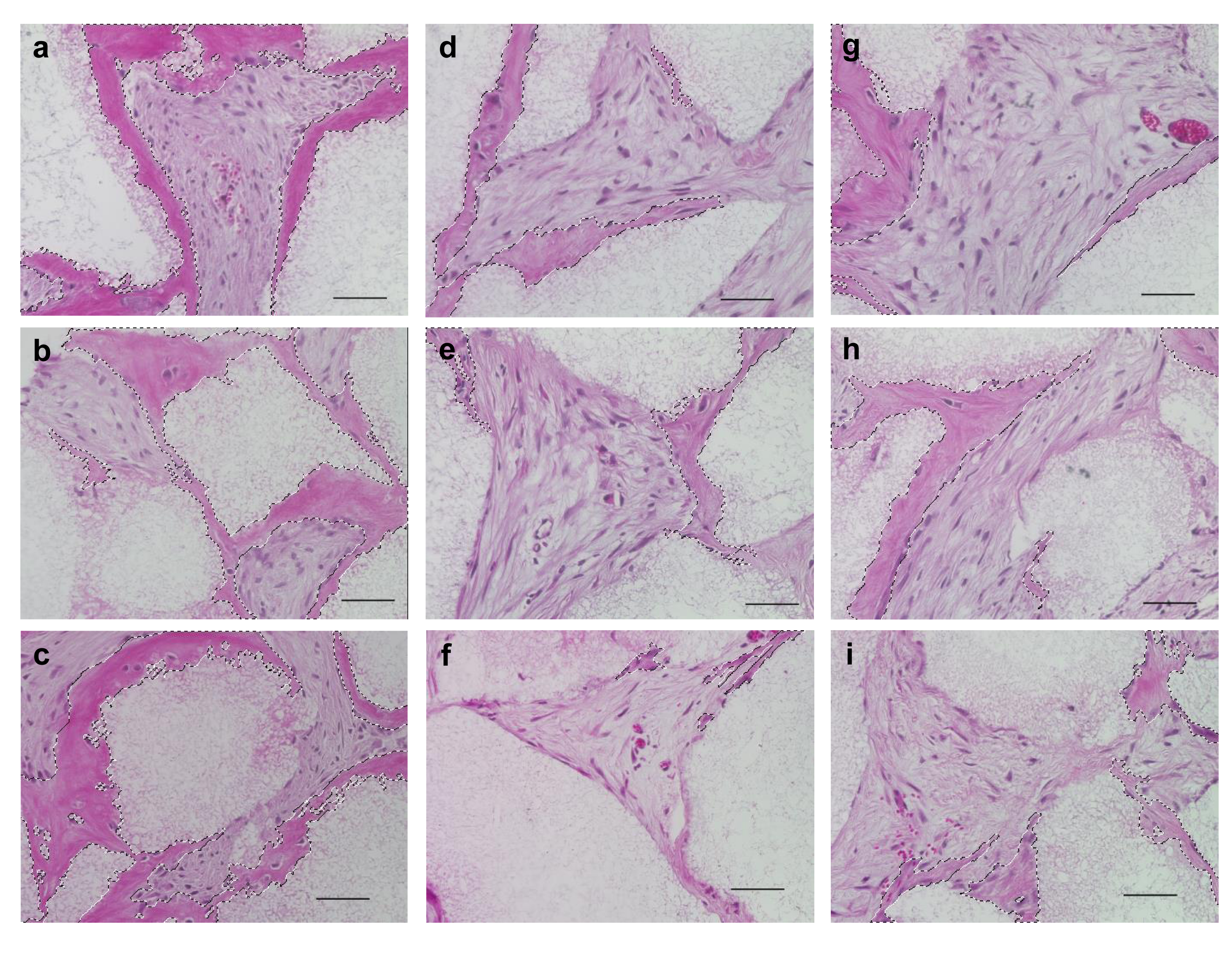

Supplement: Supplementary file 1 — Supplemental Figure S1 Dentin-like structures analysis of CD146+ (a, b, c), CD146− (d, e, f), and CD146+/− cells (g, h, i). Dotted line surrounds generated dentin-like structures area (DSA). Scale bars = 50 µm (a–i) [file 13577_2017_198_MOESM1_ESM.tif]
