# Supplementary material for: CD146 positive human dental pulp stem cells promote regeneration of dentin/pulp-like structures
Source: Hum Cell. 2018 Jan 8;31(2):127–38. doi: 10.1007/s13577-017-0198-2 (PMC5852189; doi:10.1007/s13577-017-0198-2)
Supplement: Supplementary file 2 — Supplementary material 2 (DOCX 17 kb) [file 13577_2017_198_MOESM2_ESM.docx]

**Supplemental Table S1** Proportion of dentin-like structures area (DSA) on H-E sections depicted in Supplemental Figure S1.

Sample DSA (%)

S1- a^†^ 25.9

S1- b 22.8

S1- c 28.9

S1- d^††^ 12.3

S1- e 8.7

S1- f 1.9

S1- g^†††^ 13.5

S1- h 16.2

S1- i 10.9

^†^ Measures the same image area as Fig. 5c.

^††^ Measures the same image area as Fig. 5f.

^†††^ Measures the same image area as Fig. 5i.
